# Lung protection: an intervention for tidal volume reduction in a teaching intensive care unit

**DOI:** 10.5935/0103-507X.20160067

**Published:** 2016

**Authors:** Arturo Briva, Cristina Gaiero

**Affiliations:** 1 Intensive Care Unit, Hospital de Clinicas, Universidad de la República-Montevideo, Uruguay.; 2 Area de Investigación Respiratoria, Departamento de Fisiopatología, Hospital de Clínicas - Montevideo, Uruguay.

**Keywords:** Respiration, artificial, Body weight, Lung injury, Training, Health education, Respiração artificial, Peso corporal, Lesão pulmonar, Treinamento, Educação em saúde

## Abstract

**Objective:**

To determine the effect of feedback and education regarding the use of predicted body weight to adjust tidal volume in a lung-protective mechanical ventilation strategy.

**Methods:**

The study was performed from October 2014 to November 2015 (12 months) in a single university polyvalent intensive care unit. We developed a combined intervention (education and feedback), placing particular attention on the importance of adjusting tidal volumes to predicted body weight bedside. In parallel, predicted body weight was estimated from knee height and included in clinical charts.

**Results:**

One hundred fifty-nine patients were included. Predicted body weight assessed by knee height instead of visual evaluation revealed that the delivered tidal volume was significantly higher than predicted. After the inclusion of predicted body weight, we observed a sustained reduction in delivered tidal volume from a mean (standard error) of 8.97 ± 0.32 to 7.49 ± 0.19mL/kg (p < 0.002). Furthermore, the protocol adherence was subsequently sustained for 12 months (delivered tidal volume 7.49 ± 0.54 *versus* 7.62 ± 0.20mL/kg; p = 0.103).

**Conclusion:**

The lack of a reliable method to estimate the predicted body weight is a significant impairment for the application of a worldwide standard of care during mechanical ventilation. A combined intervention based on education and repeated feedbacks promoted sustained tidal volume education during the study period (12 months).

## Abstract (pt)

**Objetivo:**

Determinar o efeito do fornecimento de comentários e educação, com relação ao uso do peso corpóreo previsto para ajuste do volume corrente em uma estratégia de ventilação de proteção pulmonar.

**Métodos:**

O estudo foi realizado entre outubro de 2014 e novembro de 2015 (12 meses) em uma única unidade de terapia intensiva polivalente universitária. Desenvolvemos uma intervenção combinada (educação e comentários), dando particular atenção à importância do ajuste dos volumes correntes para o peso corpóreo previsto ao pé do leito. Paralelamente, o peso corpóreo previsto foi estimado com base na estatura calculada a partir da altura dos joelhos e incluído nas fichas clínicas.

**Resultados:**

Foram incluídos 151 pacientes. O peso corpóreo previsto avaliado pela altura dos joelhos, em vez de avaliação visual, revelou que o volume corrente fornecido era significantemente mais elevado do que o previsto. Após a inclusão do peso corpóreo previsto, observamos redução sustentada do volume corrente fornecido, de uma média (erro padrão) de 8,97 ± 0,32 para 7,49 ± 0,19mL/kg (p < 0,002). Mais ainda, a adesão ao protocolo foi subsequentemente mantida durante os 12 meses seguintes (volume corrente fornecido de 7,49 ± 0,54 em comparação a 7,62 ± 0,20mL/kg; p = 0,103).

**Conclusão:**

A falta de um método confiável para estimar o peso corpóreo previsto é um problema importante para a aplicação de um padrão mundial de cuidados durante a ventilação mecânica. Uma intervenção combinada, que se baseou em educação e fornecimento continuado de comentários, promoveu uma redução sustentada do volume corrente durante o período do estudo (12 meses).

## INTRODUCTION

Clinicians in teaching hospitals are often early supporters of new medical advances. However, several studies recognize that while medical knowledge continues to improve, the practice of medicine continues to lag behind.^(1,2)^ The use of mechanical ventilation (MV) strategies for critically ill patients are no exception. In fact, failure to implement evidence into clinical practice is a major challenge in Critical Care.^(3)^

In a leading report, Wolthuis et al. demonstrated how a combination of educational strategies and feedback to the intensive care unit (ICU) staff can improve the quality of care for MV.^(4)^ Although our ICU staff was aware of the international recommendations for MV, we detected two main problems to solve: how to reinforce the importance of careful adjustments of tidal volume (VT) by predicted body weight (PBW) and the absence of an accurate determination of PBW itself.

Acute respiratory distress syndrome (ARDS) is a life-threatening condition that requires admission to the ICU and MV support. In addition to its severity, patients with ARDS could be injured by MV in a "second hit" called "ventilator-induced lung injury".^(5)^

In 2000, a multicenter clinical trial (ARDSNet) concluded that low delivery at 6mL/kg ideal body weight was associated with an 8.8% decrease in mortality compared with 12mL/kg.^(6)^

Despite some controversies about the best therapeutic strategy for ARDS,^(7)^ recent evidence supports the extended use of low VT even in patients without lung injury.^(8)^

In parallel to this recommendation, increasing data suggest that clinicians infrequently treat ARDS patients with a low VT strategy.^(9)^ The absence of a well-determined method to estimate the PBW appears to be a significant barrier to selecting an adequate VT. In fact, even when physicians believe they are using a low VT strategy, they may not, reflecting a protocol implementation failure.^(3)^ To determine PBW, height measurement is an essential component included in body mass index, which is not easy to measure in a critical care setting.^(10)^

Several studies have demonstrated that visual estimations of height are often inaccurate; therefore, different alternatives have been proposed.^(10-13)^

In this sense, the measurement of knee height was proposed almost 30 years ago^(14)^ to evaluate geriatric populations. At the same time, knee height is a reasonably accurate method to determine the patient´s height in the ICU.^(15)^ This method described by Chumlea et al. is rapid and easy to perform in critically ill patients,^(14)^ demonstrating fair accuracy with the patient's actual height (less than 5 cm as recommended by the World Health Organization). Following this reasoning, we included PBW in the clinical charts calculated by Chumlea's equation from knee height assessment.

The aim of this study was to determine the effect of feedback and education regarding the use of predicted body weight to adjust tidal volume in a lung-protective mechanical ventilation strategy.

## METHODS

This study was performed from October 2014 to November 2015 (12 months) in a single ICU at the *Hospital de Clinicas* (Montevideo, Uruguay), a ten bed closed polyvalent unit. Clinical decisions, including ventilator settings, are made by the medical staff, which includes five permanent members (senior and junior professors) and an additional 15 intensive care medicine specialists in rotating daily guards (assistants and residents).

**Figure 1:**
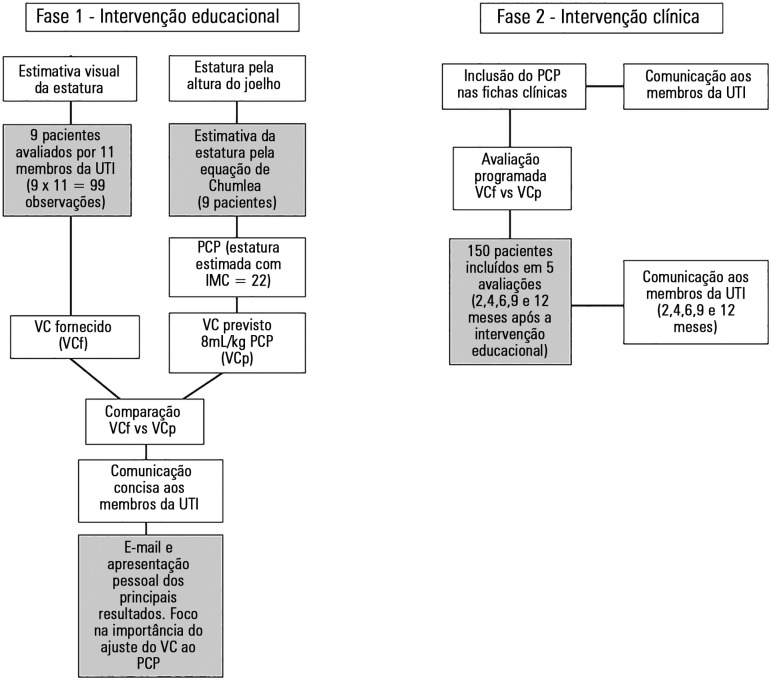
Methodological design and sample size. White boxes describe the interventions. Grey boxes add specific details. ICU - intensive care unit; PBW - predicted body weight; BMI - body mass index; VT - tidal volume.

The study was divided into two steps as is described in figure 1. Step one involved educational intervention. Nine patients receiving invasive MV were evaluated by eleven ICU members. The patient´s height was estimated by visual assessment without any other anthropometric reference. Simultaneously, real height was determined by knee height measurement and Chumlea's equation. The comparison between estimated *versus* measured height and the subsequent VT difference (delivered *versus* predicted) was quantified and communicated to the ICU staff. This feedback also included the rationale of lung-protective MV and the importance of VT adjustment by PBW.

In the second step, we evaluated whether the inclusion of PBW calculation in the clinical chart would impact delivered tidal volumes for patients on MV. This evaluation was performed at 2, 4, 6, 9 and 12 months after PBW implementation. A new feedback focused on the results obtained, and the importance of VT adjustment was e-mailed to the ICU staff for each evaluation.

**Figure 2:**
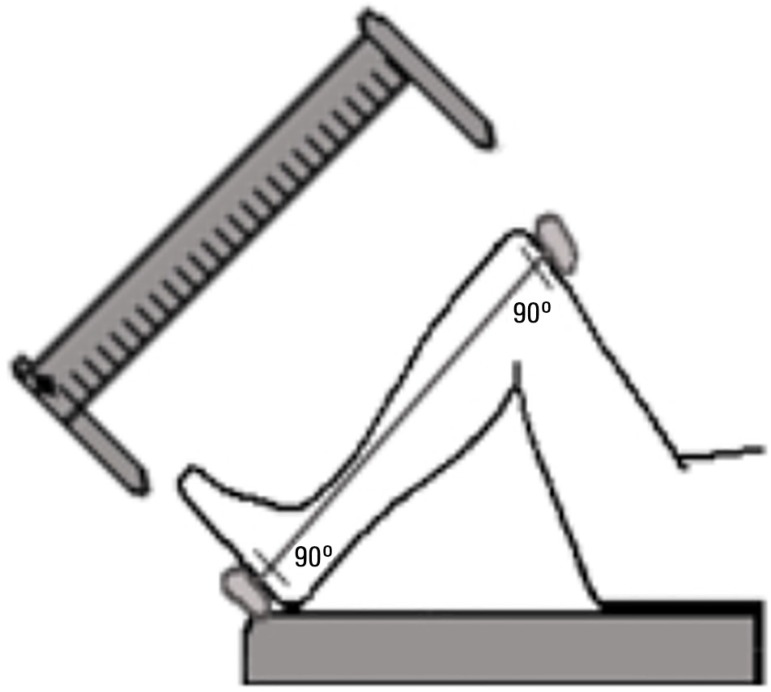
Sliding caliper schematic view and knee height measurement.

Knee height was measured with a sliding caliper as recommended by the Centers for Disease Control (http://www.cdc.gov/nchs/data/nhanes/nhanes3/cdrom/nchs/manuals/anthro.pdf) as adapted for critically ill patients.^(11)^ The measurement could be performed by a single operator while the patient is in the supine position. Both the knee and ankle of the patient should be held at a 90-degree angle (Figure 2). The fixed blade of the caliper must be placed under the heel immediately below the lateral malleolus of the fibula. In addition, the movable blade of the caliper is positioned on the anterior surface of the thigh above the condyles of the femur. The obtained knee height (in cm) is used in the Chumlea equation:

**Table:** 

Men = 64.19 - (0.04 x age) + (2.02 x knee height)
Women = 84.88 - (0.24 x age) + (1.83 x knee height)

All the patients receiving invasive MV were included and evaluated during the first 48 hours after ICU admission. Independent of the ventilator mode, we recorded the VT during 3 minutes and added the average value for the final record. Non-ventilated patients and those who died or were extubated within the first 48 hours were excluded.

Daily rounds were performed enrolling the patients within their first 48 hours on MV. An electronic data sheet was built including PBW, ideal VT, real VT and "intentional exceptions" columns. The institution's Ethics Committee approved the study protocol and the informed consent was waived.

### Statistical analysis

Data were grouped as the means and median. Standard error and maximal and minimal values were calculated. Student "*t*" test and ANOVA were performed, and a p < 0.05 was considered significant. To evaluate bias from predicted VT, we conducted a Bland & Altman analysis before and after PBW evaluation.

## RESULTS

One hundred fifty-nine patients were included in the study (nine in step 1 and 150 in step 2). The main characteristics of patients are presented in table 1. Age was not significantly different between groups, with the exception of the "9 months" group (p = 0.02). APACHE II scores were not significantly different between groups. The proportion of patients with ARDS was not statistically significant between groups (p = 0.776).

**Table 1:** Patients’ characteristics in the groups analyzed

Time (months)	Patients (number)	Age (years)	Sex (M/F)	APACHE II	Height estimated (cm)	Height measured (cm)	ARDS (%)	Mortality (%)
0	9	63.3 (6.8)	4/5	17	163 (7.6)	164 (2.1)	11.1	35.4
2	29	66.5 (1.9)	19/10	12	-	162 (2.9)	10.3	19.6
4	32	58.2 (2.5)	16/16	14	-	165 (1.8)	15.6	28.4
6	27	53.1 (3.1)	12/15	15	-	170 (3.6)	7.4	27.9
9	34	46.7 (2.8)*	21/13	13	-	161 (3.1)	20.5	28.1
12	28	60.0 (3.6)	16/12	13	-	167 (2.0)	3.5	25.2

M/F - male/female; APACHE II - Acute Physiology And Chronic Health Evaluation II; ARDS - acute respiratory distress syndrome. “0” months = initial group assessed by “visual evaluation”.

* p < 0.05 compared with time 0.

**Figure 3:**
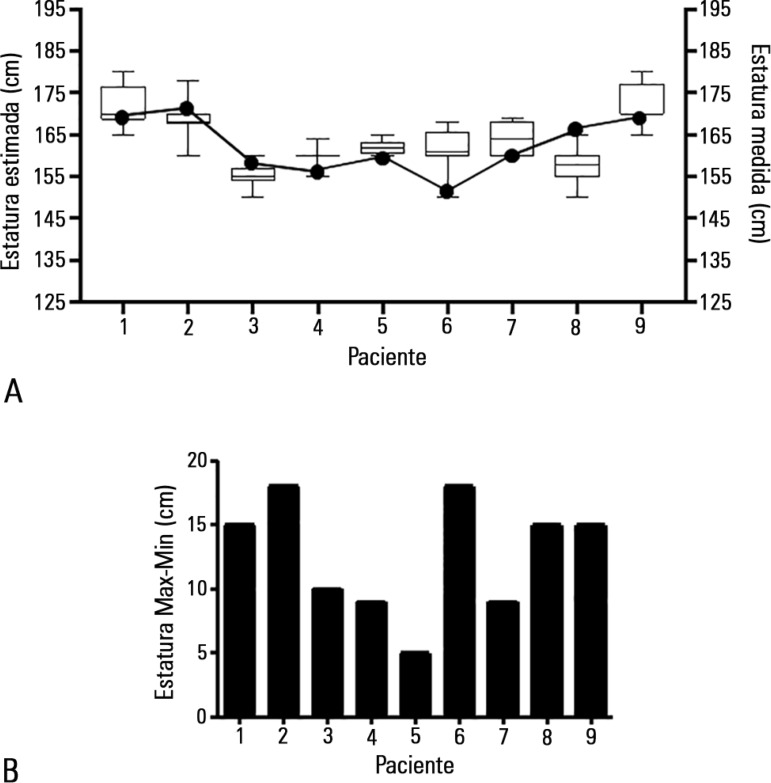
Height assessment by visual evaluation. The evaluation of nine patients by eleven observers. Box plots represent the distribution of height estimations by visual assessment, and white dots represent height determination by knee measurements (A). Graphic bars indicate the range between maximal and minimal determinations on each patient (B).

Ninety-nine observations were reported, and the results of height by visual evaluation exhibited a Gaussian distribution (D' Agostino & Pearson normality test). However, the range of values between observers varied from 9 to 15cm (Figure 3A).

Independent of the observer training and patient´s anatomy, the visual evaluation revealed a non-predictable deviation from the real height (Figure 3B).

Before the PBW adjustment, the delivered VT was significantly higher than predicted (524 ± 22.1mL *versus* 486.6 ± 16.8mL, p = 0.002) (Figure 3 and Table 2).

**Table 2:** Absolute values of delivered and predicted tidal volumes over 12 months

Time (months)	VTd (mL)	VTp (mL)	p value	VTd (mL/kg PBW)	Patients with VT > 8mL/kg PBW (%)	ARDS patients with VT > 8mL/kg PBW/Total ARDS (N)
0	524.3 (22.1)	468.6 (16.8)	0.002	8.97 (0.32)	88.8	1/1
2	465.6 (17.7)	453 (10.9)	0.569	7.49 (0.19)	20.6	1/3
4	474.3 (12.7)	441 (23.3)	0.463	7.49 (0.54)	15.6	1/5
6	510 (10.9)	522 (13.9)	0.467	7.75 (0.19)	18.5	0/2
9	466.7 (33.8)	465 (13.3)	0.954	7.78 (0.41)	26.4	1/7
12	473.1 (13.7)	497.8 (10.9)	0.103	7.62 (0.20)	17.8	0/1

VTd - delivery tidal volume; VTp - predicted tidal volume; VT - tidal volume; PBW - predicted body weight; ARDS - acute respiratory distress syndrome. “p” represents the statistical significance between delivered and predicted volumes. The delivered volume is represented also related to the predicted body weight. Values are the means and standard error.

**Figure 4:**
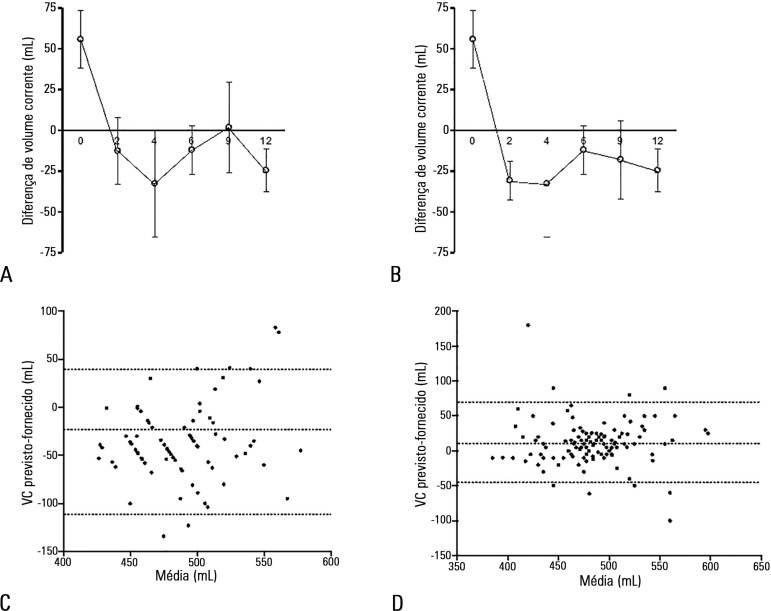
Tidal volume adjustment. The initial tidal volume delivered was significantly higher than predicted, and subsequent volumes were not significantly different during the study period. A similar pattern was observed with (A) and without (B) patients with high intracranial pressure. (Values represent the mean and standard error, * = p < 0.05). Bland & Altman plot analysis reveals a bias reduction comparing before (C) and after (D) PBW calculation. Dotted lines represent bias and 95% limits of agreement.

One hundred fifty observations were reported during the one-year study period, and the delivered VT was consistently less than 8mL/kg PBW (Table 2, Figure 4A). In the middle of the survey period, a non-significant increase in VT was detected. However, when we excluded patients with high intracranial pressure, this tendency was corrected, and the values were even lower than those initially observed (Figure 4B).

Based on Bland & Altman plots analysis of expected and delivered VT, a bias reduction was observed when comparing before and after PBW calculation (55,4 -115/+110mL *versus* 10, 41 -47/+68mL) (Figures 4C and 4D).

## DISCUSSION

After a combined intervention (education and feedback), we observed that the visual evaluation of height is inaccurate, and the delivered VT was significantly higher than predicted. However, using the PBW estimation by knee height, we observed a significant bias reduction comparing expected and delivered VT.

The translation of evidence-based knowledge to medical practice reveals problems at different levels: cognitions, motivation, working routines, team interactions and functioning of the hospital. One of the most consistent findings in behavioral research is the gap between evidence and practice.

Among several interventions developed in Critical Care to reduce the gap, there is more evidence on the combination of professional-oriented interventions (education, reminders, feedback) compared with those aimed at the organization or the patient.^(16)^

Mechanical ventilation can produce significant lung damage. Experimental^(17)^ and clinical reports^(6)^ demonstrated the relevance of tidal volume reduction to 6 to 8mL/kg of PBW as the most relevant improvement in MV practice since 2000. However, the application of reduced VT in patients under MV is an important issue worldwide.^(4,16)^ Recently, the Large observational study to UNderstand the Global impact of Severe Acute respiratory FailurE (LUNG-SAFE) reported that less than two-thirds of patients with ARDS received a VT of 8mL/kg or less of PBW.^(18)^

Our study demonstrates how a combined intervention (education and feedback) can adjust the delivered VT to patients on MV. We confirmed that visual evaluation of height is not accurate^(13)^ and promotes the underuse of lung protective strategies. On the other hand, we obtained a significant reduction in absolute values of VT after PBW implementation, with a reduction of VT bias between expected and delivered volumes (Figure 4).

In our case, the lack of a reliable PBW estimation was a significant barrier to the application of a worldwide standard of care in MV. A recently published report proposes that electronic displays with real-time metrics added to an electronic chart system reduced the delivered VT in ventilated ICU patients.^(19)^ However, our study design does not allow us to determine whether the maintenance of a lung protective strategy after 12 months is the consequence of the educational intervention, the repeated feedbacks or simply the inclusion of PBW data in the clinical chart.

A teaching ICU in a middle-income South American country is far from these technological interventions. However, we obtained similar results applying the same concept to classic paper charts and programmed feedback communications instead of using an electronic clinical record.

On the other hand, we confirmed that the estimation of patients' height by visual evaluation was highly inaccurate. This method should be strongly discouraged to calculate the PBW.

In general terms, evidence-based medicine and clinical guidelines improve the quality of health care. Moreover, deviations from guidelines increase mortality in critically ill patients.^(20)^

In recent reports, a relative increase in mortality was associated with VT violations in lung-protective strategies for ARDS^(9,21)^ and patients without lung injury.^(22)^ The use of low VT decreases ARDS development, mortality, pulmonary infections and the length of ICU stay.^(23)^ Although our study was not designed to evaluate the impact of VT reduction in clinical outcomes, the targeted VT was greater than 6mL/kg, which should be considered in the analysis as a weak point. In general, 6mL/kg is the proposed goal in several studies allowing a range from 6 to 8mL/kg based on patients' stability and comfort criteria.^(20)^ In this sense, 8mL/kg was consistently proposed as an "upper limit" in the low VT range of ventilation, which is not perfect. However, we consider this criterion as an important improvement in the quality of care.^(18)^

Independently of the previous comment, we believe that our intervention succeeds because includes three essential precepts in decision making:^(1)^ (1) the information must be ready at the time it is needed; (2) after the education event, the adjustments must be fitted into the user´s workflow; and (3) a simple intervention works better than a complex intervention.

Based on our experience, we encourage the application of educational interventions following these precepts, independent of the technological access, to improve the quality of care and teamwork for critically ill patients.

## CONCLUSION

The lack of a reliable method to estimate the predicted body weight is a significant impairment for the application of a worldwide standard of care during mechanical ventilation. A combined intervention based on education and repeated feedback promoted a sustained tidal volume reduction during the study period (12 months).
